# The Skin Microbiota and Itch: Is There a Link?

**DOI:** 10.3390/jcm9041190

**Published:** 2020-04-22

**Authors:** Hei Sung Kim, Gil Yosipovitch

**Affiliations:** 1 Department of Dermatology and Cutaneous Surgery, Miami Itch Center, Miller School of Medicine, University of Miami, Miami, FL 33136, USA; hazelkimhoho@gmail.com; 2 Department of Dermatology, Incheon St. Mary’s Hospital, The Catholic University of Korea, Seoul 06591, Korea; 3 Department of Biomedicine & Health Sciences, The Catholic University of Korea, 222 Banpo-daero, Seocho-gu, Seoul 06591, Korea

**Keywords:** itch, skin microbiota, stress, microbiota–skin–brain axis

## Abstract

Itch is an unpleasant sensation that emanates primarily from the skin. The chemical mediators that drive neuronal activity originate from a complex interaction between keratinocytes, inflammatory cells, nerve endings and the skin microbiota, relaying itch signals to the brain. Stress also exacerbates itch via the skin–brain axis. Recently, the microbiota has surfaced as a major player to regulate this axis, notably during stress settings aroused by actual or perceived homeostatic challenge. The routes of communication between the microbiota and brain are slowly being unraveled and involve neurochemicals (i.e., acetylcholine, histamine, catecholamines, corticotropin) that originate from the microbiota itself. By focusing on itch biology and by referring to the more established field of pain research, this review examines the possible means by which the skin microbiota contributes to itch.

## 1. Introduction

Bacteria, viruses, fungi, archaea, helminths, and protozoa that inhabit our body are a prospering dynamic community shaping a symbiotic superorganism. Roughly 10^14^ microbiota populate the entire body, with their number approximating that of human cells [1,2]. Evidence suggests that microbiota take part in maintaining human health [3,4].

As a crucial barrier to the exterior world, skin is one of the body’s largest organ [5]. A square centimeter of human skin holds around 10^6^ of microbiota [6,7,8]. The symbionts defend against illness by regulating the skin barrier and host immune response [9,10]. On the other hand, microbial imbalance (dysbiosis) has been noted to exacerbate skin lesions and delay wound healing [11,12]. Recently, the emerging role of the skin microbiota in itch has received attention [13].

Large-scale changes of the skin microbiota have been noted in itchy skin diseases. *Staphylococcus aureus* (*S. aureus*) participates in atopic dermatitis (AD) flare-up; its colonization correlates with disease severity and itch [14,15,16].

In the present review, we offer an integrative perspective on the skin microbiota and itch. The first section describes the interplay of the cutaneous microbiota with the epidermal barrier, the local immune system, and the sensory nerve, proposing the microbiota’s peripheral mechanism of itch. The second section concentrates on the concept of microbial endocrinology and addresses the microbiota–skin–brain axis. Moreover, the interaction between the skin microbiota and the amygdala is discussed to explain the microbiota’s central mechanism of itch. Overall, this article describes the putative role of the skin microbiota in itch.

## 2. The Peripheral Mechanism Linking the Skin Microbiota and Itch

**Figure 1:**
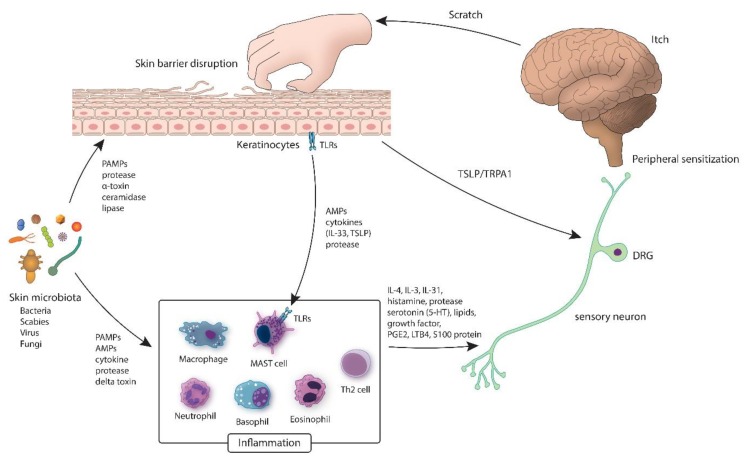
Inflammatory circuit of the skin microbiota. Various microbiota (bacteria, fungi and viruses) cover the exterior of a healthy skin where the barrier is intact. In the event of dysbiosis, pathogens release proteases, which may disrupt the epidermal barrier. Delta-toxin causes mast cell degranulation, which prompt inflammation and itching. AMP: antimicrobial peptides; DRG: dorsal root ganglia; IL: interleukin; LTB4: leukotriene B4; PAMP: pathogen associated molecular pattern; PGE2: prostaglandin E2; TLR: Toll-like receptor; TRPA1: transient receptor potential antigen 1; TSLP: thymic stromal lymphopoietin.

Itch arises from the activation of epidermal nerve fibers that belong to a specialized class of itch-provoking neurons (“pruriceptors”). The chemical mediators that drive neuronal activity arise from complex interaction between keratinocytes, inflammatory cells, and nerve endings, coupled with upregulated immune cascades, epidermal barrier dysfunction and exogenous environmental stimuli (i.e., microbiota, allergens, irritants) [17]. Peripheral nerves relay cues from the skin to the dorsal root and trigeminal ganglia and then to the spinal cord and brain where central itch processing takes place (Figure 1) [17].

### 2.1. The Skin Microbiota, The Skin Barrier, and Itch

The skin barrier shields the body from a wide range of external dangers [18]. It consists of the epidermis and several layers below that harbor microbiota [19,20,21]. The physical skin barrier is the stratum corneum, which comprises dead keratinocytes (KCs) and proteinaceous crosslinking filaments [22].

The skin also has a chemical barrier of antimicrobial peptides (AMPs) that are constitutively expressed or induced. AMPs directly block microbial growth or provoke the immune reaction. One example is the liberation of histamine and prostaglandin D_2_ (PGD_2_) [23] by mast cells in respect to human β-defensins (hBDs) and LL-37, which causes pruritus.

The skin microbiota is an integral part of the skin barrier [18]. It protects the host from pathogens by competing for nutrients and space [19]. Some produce antimicrobial compounds, which block the growth of competitors [19]. Symbionts also alter the skin barrier via bacterial enzymes, such as proteases that impact corneocyte desquamation, or lipases, which break down skin surface lipids [24].

*Staphylococcus epidermidis* (*S. epidermidis*) is the primary bacterium colonizing the human epithelia and is a vital member of the skin resident microbiota [25]. *S. epidermidis* has a flexible interrelation with its host, and deposits biofilms (a physical barrier) that are remarkably hard to clear [26]. Symbiont strains of *S. epidermidis* suppress *S. aureus* biofilm formation by producing serine protease (Esp), which also enhances the antimicrobial effect of hBD2 [27]. Another typical skin resident is *Cutibacterium acnes* (*C. acnes*) which inhibits the growth of methicillin-resistant *S. aureus* (MRSA) [28]. In short, *C. acnes* ferments glycerol, a natural metabolite in human skin, into short-chain fatty acids (SCFAs) that maintain an acidic skin pH [29]. Symbionts flourish at acidic pH, whereas potential pathogens, such as *S. aureus,* thrive at neutral pH [30,31].

Intrinsic (host) and extrinsic (environmental) factors affect skin barrier function by shaping microbial structure [32]. *S. aureus* colonization is found in up to 90% AD patients [33]. It produces ceramidase, which breaks down ceramides, an essential component of the skin barrier [34]. *S. aureus* also produces α toxins that impede wound healing and bring epithelial barrier disintegration [35].

Scabies mites (*Sarcoptes scabiei*) alter the skin microbiota by breaching the physical barrier. Epidemiologic studies in scabies patients confirmed secondary bacterial infections by two clinically important pathogens *S. aureus* and *Streptococcus pyogenes* [36].

Lately, there has been a growing awareness of fungi and their interaction with the skin barrier. When the chemical composition (i.e., sweat, pH) of the host epidermis is disturbed, Malassezia *spp.* acquire pathogenicity and liberate an array of bioactive indoles, lipases, and phospholipases [37]. These molecules further modify the function of the skin barrier.

Epithelial barrier disruption is a door opener into a vicious itch–scratch cycle [38,39]. Upon damage or stress, keratinocytes (KCs) and skin microbiota emit cytokines, AMPs, and proteases that activate immunocytes and nerves [38,40,41]. Protease-activated receptors (PARs), which are cleaved by serine proteases, manifest on different cell types, including sensory neurons and mediate itch [42,43,44,45]. β-defensin, an AMP released from epithelial cells, has the ability to stimulate IL-31 production by mast cells [46]. IL-31, initially discovered in 2004, is the first cytokine that is known to facilitate itch by directly operating on sensory neurons [47].

### 2.2. The Skin Microbiota, The Immune System, and Itch

Skin is flushed with a wide scope of cells of the innate and adaptive immune system. The skin microbiota keeps immune homeostasis [19] by modulating the expression of diverse innate factors, including AMPs, interleukin 1a (IL-1a) [48], and complement [49].

Symbionts calibrate inflammation [50,51]. *S. epidermidis* suppresses inflammation by inducing IL-10, an anti-inflammatory cytokine, from antigen-presenting cells (APCs) [52]. The Toll-like-receptor (TLR)-2-facilitated recognition of lipoteichoic acid (LTA) from *S. epidermidis* inhibits TLR-3-driven inflammatory cytokine production in cultured keratinocytes (Table 1). This also reduces inflammation in wounds, a condition where uncontrolled inflammation is damaging to the host [52].

Finally, *S. epidermidis* can finely tune the response of resident T cells and promote selective immunity against skin pathogens [57].

Alteration in the normal makeup of the skin microbiota induces inflammation. Moreover, the constitution of the cutaneous microbiota shifts dramatically in the course of inflammation [14]. For example, AD flares are tied with an overall decrease in microbial diversity with an expansion of staphylococcal species [14]. The resulting bacterial and viral infection can cause itch.

One possible mechanism of itch from *S. aureus* infection is mast cell-mediated pruriceptor stimulation. Nunez et al. discovered that *S. aureus* releases delta-toxin, an amphipathic peptide that stimulates chemical release from mast cells and mediates skin pathology in AD [58]. Serine protease from *S. aureus* is also involved in type-2 inflammation and itch [16,59].

Varicella zoster virus (VZV) causes pruritus in chickenpox by mast-cell-derived histamine [60].

KCs first detect pathogens and initiate an immune response [61]. KCs identify an array of microbial ligands via Toll-like receptors (TLRs) exhibited on their surface [62,63,64]. In response to stimulation, KCs produce alarmins or epithelial cell-derived cytokines (i.e., IL-33, thymic stromal lymphopoietin (TSLP)) [65] which potentiate innate and adaptive immunity [61]. TSLP also acts upon a subdivision of TRPA1 sensory neurons to spark itch [65].

Mast cells (MCs) are also an essential element of innate immunity. MCs recognize pathogens via pathogen-associated molecular pattern (PAMP) receptors (i.e., TLR) on their surface [66]. Once they detect pathogens, inflammatory mediators are released to attract other immune cells [67,68].

Downstream of IL-33 and TSLP, mast cells, neutrophils, basophils, eosinophils, Th2 cells, and macrophages generate cytokines (IL-4, IL-13, IL-31), histamine, proteases, serotonin (5-HT), lipids, S100 proteins, prostaglandin E2 (PGE2), leukotriene B4 (LTB4), and growth factors [69,70,71]. Recognizing these pro-inflammatory molecules via TRPV1 and TRPA1 channels leads to action potential propagation across the afferent itch pathway [72].

Th2 immunity is dominant in scabies and is complemented by a heavy inflow of IL-31(+) M2 macrophages [73]. Proteases from scabies mite stir epidermal KCs to express TSLP. TSLP activates Th2 cells and induces M2 macrophages to produce IL-31, causing severe itch [74]. The antigens of *S. aureus* have also been reported to induce IL-31 in individuals with AD [75].

### 2.3. The Skin Microbiota, The Sensory Nerve, and Itch

Skin is one of the first lines of defense against microbial threats. Though the immune system is an essential component of cutaneous immunity, it is evident that the sensory nervous system also plays an important part in host defense. By evoking the sensation of itch, the host can immediately sense danger and rapidly initiate a protective behavioral response [69].

**Figure 2:**
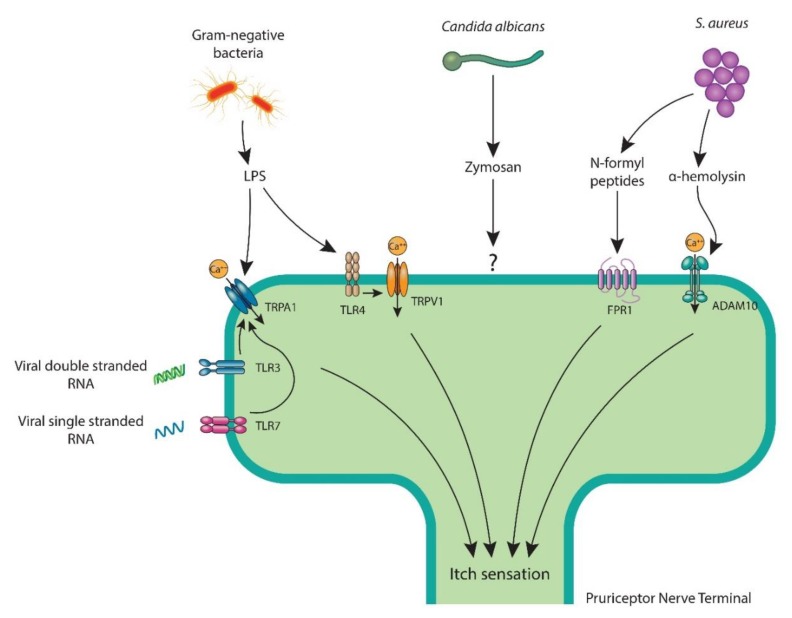
Pruriceptor neurons recognize skin pathogens and their molecular ligands by various mechanisms to facilitate itch. LPS, a key cell wall component of Gram-negative bacteria attaches to neuronal TLR4 and primes TRPV1 ion channel or opens the TRPA1 ion channel. *S. aureus* triggers itch with bacterial N-formyl peptides that bind to FPR1 or via α-hemolysin, which couples with ADAM10. *C. albicans* stimulates pruriceptors with its cell wall component zymosan. Viral double-strand RNA and single-strand RNA bind to TLR3 and TLR7, respectively, which are believed to sensitize the TRPA1 ion channel. ADAM10: a disintegrin and metalloproteinase domain-containing protein 10; FPR1: formyl peptide receptor 1; LPS: lipopolysaccharide; RNA: ribonucleic acid; TLR: Toll-like receptor; TRPA1: transient receptor potential ankyrin 1; TRPV1: transient receptor potential vanilloid 1.

A network of high- and low-threshold sensory nerves innervates the skin and is frequently exposed to bacterial pathogens (Figure 2).

Pruriceptor neurons express cytokine receptors and G protein-coupled receptors that recognize immune mediators [76]. While we understand that microbial inflammation propagates itch, how the skin microbiota directly triggers sensory nerves is a new area of inquiry.

The latest studies suggest that sensory neurons, like immune cells, are able to detect microbiota [13,69,76,77]. Ji and colleagues reported TLR7 on pruriceptors and noted synthetic TLR7 ligands (i.e., imiquimod) causing itch behavior in mice [78]. TLR3 is also displayed by pruriceptors, where Polyl:C, a TLR3 ligand, stimulates neuronal activity and itch [79]. Viral single-stranded RNA and double-stranded RNA are known pathogen-derived ligands for TLR7 and TLR3, respectively, and there is a possibility that these viral ligands cause itch by directly interacting with pruriceptor neurons [76].

Lipopolysaccharide (LPS), an important component of the Gram-negative bacteria outer membrane, binds to TLR4 [80]. Although LPS has only been reported with pain [81], it can also modulate itch since TLR4 promotes histamine-mediated itch [82]. Interestingly, LPS has also been found to stimulate sensory neurons in an TLR4-independent manner, via the activation of TRPA1 [83,84].

Besides TLR ligands, sensory neurons notice pathogens through various molecular means. Specifically, zymosan from *Candida albicans* [85], N-formylated peptides and α-hemolysin from *S. aureus* [86], and streptolysin S from *S. pyogens* [87] were shown to mediate pain through direct neuronal stimulation. It remains to be discovered whether pruriceptors recognize these pathogens in a matching manner to elicit itch.

Itch is bothersome in patients with cholestatic liver disease [88]. Recently, alteration of the skin microbiota was identified in cirrhosis patients where specified microbial taxa correlated with itch severity and serum autotaxin (ATX) level [89]. Lysophosphatidic acid (LPA), a powerful neuronal activator, and ATX (ectonucleotide pyrophosphatease/ phosphodiesterase 2), the enzyme that creates LPA, are pruritogens in cholestasis [90,91]. It is suggested that LPA directly activates TRPV1 on peripheral sensory neurons to mediate itch [92].

Neuroimmune conversation is bidirectional in itch. Sensory neurons are sensitized by immune cell-made cytokines (i.e., TNF-α, IL-1β), chemicals (i.e., histamine), and lipid mediators (i.e., prostaglandins), which phosphorylate ion channels and lower the bar of action potential firing. Neurons, in turn, secrete neuropeptides (i.e., calcitonin gene-related peptide, substance P) that modulate immune cell function [93,94] and microbial virulence [95,96,97] causing itch [98]. As neurons respond within milliseconds of facing danger, the sensory nervous system is likely the first to notice pathogen invasion and the prime orchestrator itch [76].

## 3. The Central Mechanism Linking the Skin Microbiota and Itch

### 3.1. Microbial Endocrinology

Microbial endocrinology is a crossing of two supposedly distinct areas, microbiology and neurobiology, and is based on the shared presence of neurochemicals in the host and the microbiota [66].

The scope of neurochemicals and the variety of microbiota in which they are discovered is huge [99]. These include acetylcholine [100,101], histamine [102,103], serotonin [104], catecholamines [105,106], and agmatine [107,108], which are essential elements of an animal’s nervous system. Others, such as corticotropin [109], somatostatin [110], and progesterone [111], have biological action in mammalian cells.

The ability of the microbiota to not only respond to but also create the very same neurochemicals of mammalian systems, tells that host interplay with the microbiota is much more interactive than it was thought before.

Hence, microbial endocrinology could be applied beyond infectious disease to other conditions such as brain health through the microbiota–skin–brain axis.

Microbiota has multiple transmission pathways to access the brain: the neural signals carried by the afferent neurons, endocrine messages transmitted by neurochemicals and the immune messages transferred by cytokines [112,113].

The skin is one important platform for microbial communication with the brain. In an evolutionary standpoint, it is reasonable for the skin to support the cutaneous microbiota, which in turn assists skin barrier function and local immune system and helps the skin communicate with other organ systems, including the brain (microbiota–skin–brain axis) [114].

### 3.2. Stress, The Skin Microbiota, and Itch

Stress is a complex dynamic condition where homeostasis, or the stability of an organism is altered, promoting the adaptation of the host. Stress aggravates itch [115,116,117], which proves that the brain is engaged in the final common stage of itch processing [118,119].

Stress acts by the central nervous system (CNS) and alters the microbiota via the release of neurochemicals [120,121]. Glucocorticoids, an essential component of the stress response, repress AMP release/localization in the epidermis, weaken the barrier, and raise host susceptibility to infection [122,123,124].

**Figure 3:**
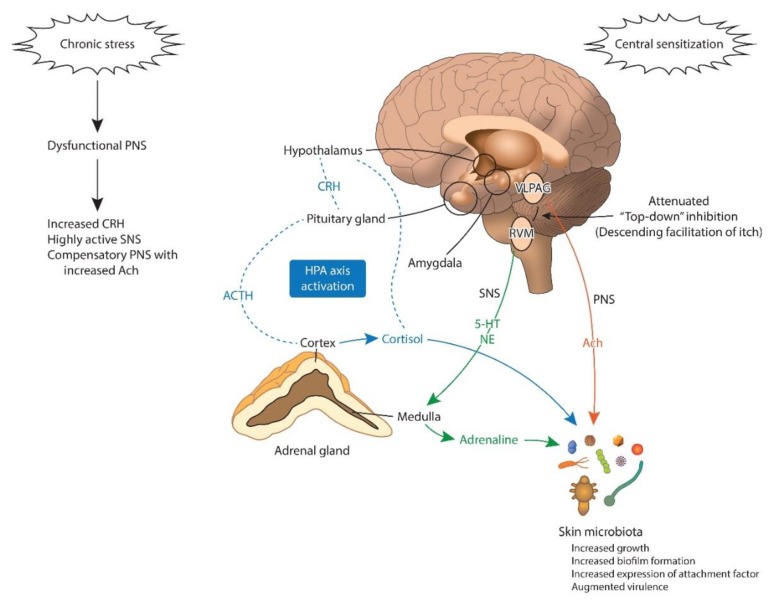
Brain to microbiota communication under chronic stress. The HPA axis is activated under chronic stress. The final product of the HPA axis, cortisol, directly activates skin microbes. Cortisol activates the amygdala, promoting central sensitization to itch. The amygdala also promotes CRH signaling to the brain stem (PAG), altering the “descending itch modulatory system”. Prolonged exposure to cortisol, NE, and ACTH is associated with increased growth and biofilm genesis and augmented virulence of the skin microbiota. Ach: acetylcholine; ACTH: adrenocorticotropic hormone; CRH: corticotropin-releasing-hormone; HPA: hypothalamic–pituitary–adrenal axis; 5-HT: serotonin; NE: norepinephrine; PNS: parasympathetic nervous system; RVM: rostral ventromedial medulla; SNS: sympathetic nervous system; VLPAG: ventrolateral periaqueductal grey matter

Chronic stress is associated with an aberrant parasympathetic tone (Figure 3) [125,126]. Cholinergic signaling from physiologic stress [125] negatively impacts the skin barrier and immunity [127,128]. Cathelicidin and β-defensins, AMPs important for innate immunity, were cut down after α7nAChR stimulation [128,129], leading to bacterial dissemination.

Skin microbiota, especially the coagulase-negative staphylococci, are sensitive to catecholamines. Norepinephrine (NE), epinephrine, dopamine, and their structurally related inotropes (dobutamine and isoprenaline) raise staphylococcal growth by 5-log orders or more [130,131,132]. Catecholamines also strengthen bacterial attachment to host tissues and increase bacteria virulence [130,133,134]. Catecholamines stimulate the biofilm formation of *Pseudomonas aeruginosa* (*P. aeruginosa*) and *Escherichia coli* (*E. coli*) [124,135]. Within a polymirobial biofilm, *P. aeruginosa* enhances USA300 methicillin-resistant *S. aureus* virulence [136].

Substance P is released in sweat during stress and increases the virulence of Gram-positive skin bacteria, namely *S. aureus* and *S. epidermidis* [95,96].

Thus, the effect of stress on the skin microbiota may be twofold: dampening the host defense to infection and adjusting the microenvironment ideal for pathogens [124]. The resultant dysbiosis can exacerbate itch (“stress aggravated itch”) (Table 2).

### 3.3. The Skin Microbiota, The Amygdala, and Itch

Itch encompasses sensory-discriminative and affective-motivational aspects and undergoes extensive processing in the higher brain centers [119,125].

The Amygdala is involved in pain, especially in the emotional-affective aspects of pain perception [144]. The central nucleus of the amygdala (CeA) is commonly called the “nociceptive amygdala” [145] and receives peripheral pain signals via the parabrachial nucleus [146].

The role of amygdala in itch is also shown in animal studies [147]. A recent study noted that scratching was suppressed after blocking itch-mediating spinal neurons connected to the spinoparabrachial pathway [148]. Additionally, an animal functional MRI (fMRI) study presented amygdala activation during itch stimuli [149]. The findings hint that itch signals are delivered by both the spinothalamic pathway and the spinoparabrachial-CeA path. It was claimed that the injection of muscimol (γ-aminobutyric acid agonist) to the amygdala minimized scratching elicited by the injection of serotonin to the cheek, implying a modulatory role of the amygdala in itch processing [150].

Chronic stress brings functional and configurational changes in the amygdala (central sensitization) (Figure 3) [151]. This change may influence itch processing in the brain, which explains why stress worsens itch in individuals with chronic itch [152,153].

Studies suggest that the amygdala itself is susceptible to microbial influences [154]. Most convincingly, data from germ-free (GF) mice imply that the amygdala transcriptome becomes hyperactive in the absence of microbiota [155,156]. This hyperactive state is in line with the altered pain sensitivity [157] and stress response in GF mice [158,159].

We do not know how microbial signals navigate through the skin–brain axis to reach the amygdala specifically yet; however, there are some strong candidate paths, including the blood stream (circulation) and the spinal cord [112,154,160].

## 4. Conclusions and Future Perspectives

With increased recognition of the presence and functionality of the microbiota, the human body is not what we perceive. Evidence suggests that our microbiota occupies a prominent role in the human body than formerly thought.

Cutaneous microbiota delivers a diverse and far-reaching influence on our physiology by calling upon the host nervous system. Bacteria make metabolites, toxins, and structural components that are recognized by peripheral and central neurons via matching receptors. Microbiota also indirectly affects neural function by causing endocrine (i.e., keratinocytes) and immune cells to transmit signals (i.e., cytokines, proteases). Itch is a prototypic sensory neural function, and the microbiota propels the itch–scratch cycle.

Some descriptive studies have differentiated the microbiota found in itchy skin versus those of healthy skin. While dysbiosis is found in various pathologies, these raise a “chicken-or-the-egg” type question, as we are not sure if dysbiosis leads to disease, or whether the underlying conditions cause microbial imbalance.

To differentiate cause and effect, a deeper and more mechanistic (functional) understanding of the skin microbiota’s role in itch is required. Increased grasp of this area will help find microbiological markers in itchy conditions and develop alternative therapeutics which utilize host–microbiota relationship.

The gut and skin are uniquely related in function, and numerous studies link gut microbiota to skin homeostasis (skin–gut axis or skin–gut–brain axis) [35,161,162,163,164]. Commonalities have also been found between itch transition in the skin and neural signaling in the lower intestinal tract, which raises the possibility of intestinal microbiota playing a role in itching [165,166].

**Figure 4:**
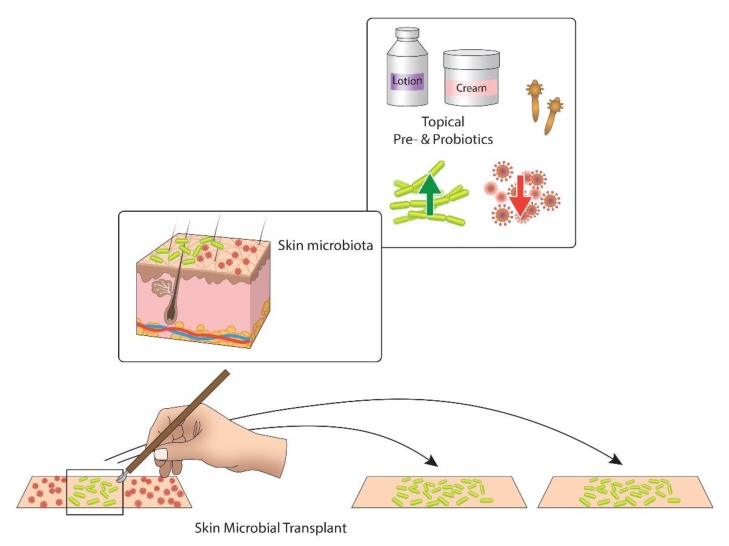
Two main approaches of controlling the human skin microbiota for the itch control. Topical pre- and probiotics target to increase the number of advantageous bacteria (green) and reduce pathogens (red). Skin microbial transplant is a new approach that transfers beneficial microbiota from healthy skin to itchy and dysbiotic skin [167].

Various types of microbiota-based therapy may be applied in the upcoming years: (1) Whole microbiota transplant, a process that offers microbiota from healthy donors to patients with significant skin dysbiosis, such as AD, to correct the dysbiosis. The favorable effects of microbial transplant can be studied for other itchy conditions as well. (2) Topical probiotics can be used to introduce known advantageous microbiota to a patient (especially at a critical age for immune and limbic brain wiring). (3) Topical prebiotics (nutrients that stimulate beneficial skin micrbiota, or biomass or dead extracts of non-pathogenic bacteria which antagonize substance P) can be adopted. (4) Host–microbiota interplay can be studied by analyzing microbial metabolites, re-imposing commensal microbial activity by offering signaling molecules (Figure 4).

In conclusion, the interplay between the skin microbiota and itch is an emerging area to explore. In future, cosmetics/transdermal drugs with a concept of ‘topical microbiota modulator’ could have the potential to claim that they do not only make you look good but also that make you feel good.

## Figures and Tables

**Table 1:** Interaction between the skin microbiota and the Toll-like receptors (TLRs).

Bacteria	Interactions with TLRs
*S. epidermidis*	Adjusts TLR3-dependent inflammation by introducing a TLR2-mediated crosstalk to subdue inflammation [52]. Elicits keratinocytes to display AMPs through a TLR2-dependent mechanism [50].
*S. aureus*	Induction of hBD3 gene expression is TLR2-dependent [53]. Lipoteichoic acid and bacterial lipoproteins act as TLR2/2 or TLR2/6 agonists [54,55].
*P. acnes*	Colonizes sebaceous glands and stimulates KCs to release inflammatory cytokines via TLR2 activation [56].

**Table 2:** Effects of stress mediators on the skin microbiota.

Bacteria	Effects of Stress Mediators
*Staphylococcus epidermidis*	Glucocorticoids decrease the effects of super antigen activated T cells and inhibit staphylococcal exotoxin-induced T cell proliferation, cytokine secretion [137]. Catecholamines induce biofilm growth [130].
*Propionibacterium acnes*	Cortisol and steroids significantly exacerbate inflammation associated with *P. acnes* via TLR2 stimulation [138,139].
*Pseudomonas aeruginosa*	Norepinephrine increases expression of the attachment factor PA-1 of *P. aeruginosa* and increase biofilm formation [135,138].
*Staphylococcus aureus*	Acetylcholine augments susceptibility to infection by *S. aureus* [124]. Norepinephrine increases *S. aureus*’ ability to remove iron from host and therefore facilitates the bacteria to form biofilms [138,140].
*Group A Streptococcus*	Cortisol alters vulnerability to *Group A Streptococcus pyogenes* skin infection [141]. Acetylcholine augments susceptibility to infection by Group A Streptococcus [124]. Catecholamines raise Staphylococcal growth by 5-log orders [130,131,132]. Catecholamines enhance *Group A Streptococcus* growth likely by increasing iron availability [138,142].
*Candida*	Estrogen enhances *Candida* infectivity, switching yeast form to an invasive hyphae [143].

## Glossary

**Term**	**Definition**
16S rRNA gene sequencing	Genomic analysis of 16S ribosomal RNA phylotypes from DNA that is extracted directly from bacterial communities in clinical or environmental samples, a process that circumvents culturing [29].
Skin microbiota	Total of microbes in/on our skin [168].
Microbiota	The group of microbes found in/on a specific environment or living host [169].
Microbial diversity	Degree of variability of the microbiota. α-diversity describes within-sample variability, while β-diversity signifies variability between samples [169].
Dysbiosis	Microbial imbalance or maladaptation [168].
Prebiotics	Nutrients that stimulate beneficial skin microorganisms [170].
Probiotics	Live microorganisms that have a favorable impact on host health when given in proper amounts [169].
Antibiotics	Antibiotics block the growth of or destroy bacteria and other microbes [168].
